# Development and validation of the kindergarten teacher job crafting scale in China

**DOI:** 10.1186/s40359-026-04925-4

**Published:** 2026-06-11

**Authors:** Xiaoqing Lin, Runkai Jiao, Feifei Li

**Affiliations:** 1 School of Education and Psychology, Minnan Normal University, 36 Xianqian Zhi Street, Zhangzhou, Fujian Province 363000 China; 2 Fujian Province Key Laboratory of Applied Cognition and Personality, Zhangzhou, China; 3 School of Psychology, Northeast Normal University, 5268 Renmin Street, Changchun, Jilin Province 130024 China; 4 College of Education, Center for Psychological and Behavioral Research, Wenzhou University, Chashan Higher Education Park, Wenzhou, Zhejiang 325035 China

**Keywords:** Job crafting, Scale development, Chinese kindergarten teachers, Validity, Reliability

## Abstract

**Background:**

Job crafting is important for kindergarten teachers' professional adaptation and well-being. However, existing measures may not adequately reflect context-specific manifestations in kindergarten settings. Grounded in the role-resource integration perspective, this study aimed to develop and validate a scale for assessing kindergarten teachers' job crafting.

**Methods:**

In Study 1, semi-structured interviews were conducted with 43 kindergarten teachers to generate an initial item pool. In Study 2, a convenience sample of 1,224 kindergarten teachers was randomly divided into two independent subsamples. Sample 1 was used for item analysis and exploratory factor analysis. Sample 2 was used for confirmatory factor analysis and for testing reliability and validity. Measurement invariance was examined using a full sample.

**Results:**

In Study 1, interviews and qualitative analyses identified the core elements of kindergarten teachers' job crafting and generation of informed items. In Study 2, exploratory factor analysis yielded a final scale comprising 16 items for approach job crafting and 13 items for avoidance job crafting. Each subscale included three dimensions: task crafting, relational crafting, and cognitive crafting. Confirmatory factor analysis supported the second-order structure of the subscales. Both subscales demonstrated good reliability, convergent validity, discriminant validity, and criterion-related validity and showed acceptable measurement invariance across groups.

**Conclusion:**

The newly developed Kindergarten Teacher Job Crafting Scale demonstrates good psychometric properties. This is a reliable tool for research and practice in kindergarten management and professional teacher development.

## Introduction

Kindergarten teachers play a critical role in delivering high-quality early childhood education [45]. Their work is characterized by high emotional demands and fragmented workloads, as pedagogical responsibilities intertwine with caregiving, emotional support, behavioral guidance, and frequent parent–teacher interactions [12, 22, 23, 28, 32, 35]. Relatively low compensation further contributes to challenges related to career stability, professional development, and turnover intention [20, 26, 42]. Job crafting has been identified as a proactive way for teachers to improve their work experience and manage complex job conditions, [5, 51]. Empirical studies have shown that job crafting is associated with a range of positive outcomes among kindergarten teachers, including work engagement [1, 51], job satisfaction [5], organizational commitment and quality of care and education [15], and job performance [51]. These findings highlight the importance of clarifying the conceptual meanings and specific manifestations of job crafting among kindergarten teachers.

Job crafting is a self-initiated process through which individuals modify their work [3]. Research has developed from two perspectives: role and resource. The role perspective emphasizes redefining job boundaries to enhance work meaning and shape work identity, encompassing task, relational, and cognitive crafting [43]. In contrast, the resource perspective, grounded in the Job Demands-Resources model, focuses on adjusting job demands and resources to improve work functioning and person-job fit, typically involving increasing structural and social resources, increasing challenging demands, and decreasing hindering demands [36–38]. Although both perspectives emphasize proactive change, they differ in conceptual focus and operationalization, contributing to fragmentation in the theoretical development of job crafting [3, 48].

To address fragmentation across theoretical perspectives, recent research has proposed an integrated approach grounded in approach-avoidance theory. From this perspective, job crafting is conceptualized as approach job crafting (AJC) and avoidance job crafting (AvJC) [3, 8, 19, 24, 49]. AJC is oriented toward attaining desirable work-related states and is typically manifested in expanding, enriching, or improving aspects of work. In contrast, AvJC is oriented toward reducing undesirable conditions and is typically manifested in limiting or minimizing the sources of strain. AJC and AvJC are related but relatively independent constructs that may coexist within individuals, rather than forming opposite ends of a single continuum [19, 31]. Within this framework, Zhang and Parker [49] proposed a hierarchical model that differentiates job crafting by motivational orientation (approach and avoidance), form (behavioral and cognitive), and content (resources and demands). Although this model offers an integrated framework, its structural complexity presents measurement challenges. In particular, conceptualizing cognitive crafting primarily in terms of job resources and demands may obscure the meaning-related functions emphasized in the role perspective. Moreover, the boundary between job resources and demands is often ambiguous in practice, further complicating classification and application [48]. These conceptual ambiguities and operational challenges highlight the need for more precise instruments for measuring job crafting.

Job crafting is widely regarded as occupation-specific, and its forms and content may vary across work contexts [25, 27]. Therefore, general job crafting measures may have limited applicability in specific occupational settings, whereas context-sensitive measures may better capture actual job crafting behaviors [2]. This limitation is particularly salient for kindergartens in China. Teachers' work is shaped by distinctive cultural and professional conditions, including an emphasis on interpersonal harmony, intensive emotional labor, and frequent parent–teacher interactions [10, 14, 47]. Consequently, general measures may not fully capture the structures and specific manifestations of job crafting among Chinese kindergarten teachers.

Existing research on kindergarten teachers' job crafting has primarily focused on role and resource perspectives. From the role perspective, research has focused mainly on task crafting [15], with relatively limited attention paid to the relational and cognitive dimensions. Subsequent qualitative research suggests that job crafting among kindergarten teachers extends beyond task adjustments to include interpersonal interactions and professional meaning [51]. From the resource perspective, most studies have adapted measures developed in general occupational contexts (e.g., [38]), which may not fully capture the specific characteristics of kindergarten work. Recent research has also begun to examine job crafting from an integrated perspective. Within the integrated perspective, job crafting has been conceptualized in terms of approach and avoidance orientations, behavioral and cognitive forms, and job resources and demands [18]. However, this hierarchical structure remains difficult to operationalize clearly in context-specific settings such as kindergarten teaching. Overall, existing measurement instruments remain limited in clearly distinguishing the different forms of job crafting and capturing their contextualized expressions. Therefore, a context-sensitive measurement structure grounded in an integrated perspective is needed to capture kindergarten teachers' job crafting more accurately.

This study aimed to develop and validate a contextually grounded and theoretically integrative job-crafting measure for Chinese kindergarten teachers. Job crafting was conceptualized as comprising both AJC and AvJC, each including behavioral and cognitive forms. The research comprised two studies. Study 1 employed semi-structured interviews and content analysis to examine contextualized expressions of job crafting and inform item development. Study 2 refined and validated the Kindergarten Teachers' Job Crafting Scale (KTJCS). The criterion-related validity was examined based on work engagement, work meaning, and job satisfaction.

## Study 1

Study 1 employed a qualitative design to examine kindergarten teachers' job crafting in daily practice from an integrated perspective and to generate context-based descriptions for subsequent item development.

## Method

### Participants and procedure

The interview sample was recruited through purposive sampling supplemented by snowball sampling. A total of 43 kindergarten teachers participated voluntarily without compensation. Participants were drawn from four provinces in China (Jilin, Shandong, Heilongjiang, and Jiangxi), selected to represent diverse socioeconomic and educational contexts. The sample included 25 teachers from public kindergartens (58.14%) and 18 from private kindergartens (41.86%). Participants had a mean age of 31.60 years (*SD* = 5.30; range = 22–46) and an average teaching experience of 9.19 years (*SD* = 5.80; range = 2–27). All participants were directly engaged in daily educational practice with children.

Semi-structured interviews were conducted to explore kindergarten teachers' understanding of and experiences with job crafting. Depending on participants' availability and geographic location, interviews were conducted either face-to-face or via online voice calls, each lasting approximately 40 to 60 min. The interview guide was developed based on existing conceptualizations of job crafting and informed by previously validated interview frameworks (e.g., [3, 51]) to ensure systematic data collection relevant to the research questions.

At the beginning of each interview, participants were invited to describe their daily work tasks, role responsibilities, and broader perceptions of their professional role to establish contextual grounding. Then, participants were provided with a brief explanation of job crafting to ensure a shared understanding of the interview focus. They were asked to recall specific proactive adjustments they had made in their work, including efforts aimed at enhancing positive work experiences as well as reducing stress or negative experiences. Follow-up questions were used as needed to further explore the contexts and enactment of these adjustments. Participants were not required to respond according to predefined categories. Data collection and analysis proceeded concurrently until theoretical saturation was achieved, as reflected by a substantial decline in newly identified codes and the absence of meaningful new themes. All interviews were audio-recorded with participants' informed consent and transcribed verbatim for subsequent analysis.

### Analytic strategy

This study drew on the coding procedures of grounded theory and used QSR NVivo 12.0 to analyze interview data on kindergarten teachers' job crafting. Specifically, open coding, axial coding, and selective coding were conducted sequentially. During open coding, interview transcripts were reviewed line by line to identify initial concepts. During axial coding, conceptually similar codes were compared, refined, and integrated into higher-order categories, and the relationships among these categories were examined. During selective coding, core categories were further integrated and refined to develop the conceptual framework underlying kindergarten teachers' job crafting. The resulting categories were subsequently compared with existing conceptualizations of job crafting to refine the theoretical structure and inform subsequent item development.

## Results

To enhance the credibility of the coding results, the interview data were collaboratively coded by two coders: the researcher and a doctoral student in psychology with relevant expertise. Both coders carefully reviewed the original interview data and engaged in repeated comparisons, discussions, revisions, and trial coding before arriving at the final coding scheme. Discrepancies were resolved through discussions until a consensus was reached. Inter-coder reliability was assessed using a category agreement index. Across the 43 interviews, agreement coefficients ranged from 0.827 to 0.951, indicating satisfactory coding consistency.

Interview data indicated that both AJC and AvJC manifested through behavioral and cognitive crafting. Cognitive crafting emerged as a relatively unified theme, whereas behavioral crafting was further differentiated into task and relational crafting. AJC and AvJC were ultimately represented by three themes: task, relational, and cognitive crafting. For AJC, task crafting was reflected in efforts to improve teaching tasks; relational crafting in actively shaping interactions with colleagues, children, and parents; and cognitive crafting to positively reframe the meaning of work. For AvJC, task crafting was reflected in reducing stressful tasks, relational crafting in minimizing strain-inducing interactions, and cognitive crafting in avoiding a deeper reflection on the negative aspects of work.

The initial items were generated from semi-structured interviews with kindergarten teachers and supplemented with a review of existing job crafting scales. Although the interview guide was informed by existing conceptual definitions, the coding process prioritized participants' original expressions and emergent meanings to ensure that the final thematic structure remained grounded in their work experiences. Items from existing scales were adapted only when conceptually relevant and appropriate in the kindergarten teaching context. The two sources were then integrated to form an initial item pool of 34 AJC and 25 AvJC items. Content validity and item appropriateness were evaluated by six psychology faculty members, two doctoral students in psychology, and one doctoral student in early childhood education. They assessed the items for clarity, comprehensibility, and consistency with conceptual definitions of job crafting. Based on their feedback, several items were merged, deleted, or reworded, resulting in a refined questionnaire comprising 27 AJC and 21 AvJC items.

## Study 2

Building on the qualitative findings of Study 1, Study 2 aimed to refine and validate a multidimensional measure of Chinese kindergarten teachers' job crafting by examining its factor structure, reliability, construct validity, measurement invariance, and criterion-related validity regarding work engagement, work meaning, and job satisfaction. The following hypotheses were proposed based on the theoretical framework and Study 1 results.

- Hypothesis 1: The KTJCS comprises AJC and AvJC subscales, each including task, relational, and cognitive crafting.
- Hypothesis 2: AJC is positively associated with work engagement, work meaning, and job satisfaction, whereas AvJC is negatively associated with these outcomes.

## Method

### Participants and procedure

A cross-sectional survey was conducted using convenience sampling across multiple kindergartens in Guangdong and Jilin provinces. Located in southern and northern China, respectively, these two provinces represent regions with different levels of economic development, thereby enhancing regional diversity within the sample. All participants were full-time kindergarten teachers, ensuring occupational homogeneity and supporting the suitability of the sample for scale development. Data were collected anonymously via the Wenjuanxing online platform. Before the formal questionnaire began, an informed consent statement was presented, emphasizing voluntary participation, anonymity, confidentiality, and the academic purpose of the study. Participants who provided digital informed consent were permitted to proceed to the questionnaire. This study was approved by the Ethics Committee of Northeast Normal University, and all procedures were conducted in accordance with the Declaration of Helsinki.

A total of 1,459 responses were initially collected. After excluding invalid questionnaires due to repetitive response patterns or excessive missing data, 1,224 valid responses were retained, yielding an effective response rate of 83.89%. The full sample was then randomly divided into two independent subsamples. Sample 1 was used for item analysis and exploratory factor analysis (EFA). Sample 2 was used for confirmatory factor analysis (CFA), validity testing, and reliability analysis. Measurement invariance was examined using the full sample.

Sample 1 comprised 612 kindergarten teachers, with a mean age of 35.51 years (*SD* = 8.19; range = 20–55) and a mean teaching experience of 11.45 years (*SD* = 8.58; range = 1–40). Regarding educational attainment, 38.70% (*n* = 237) held a junior college degree or lower, and 61.30% (*n* = 375) held a bachelor's degree or higher. In terms of kindergarten type, 84.60% (*n* = 518) worked in public kindergartens and 15.40% (*n* = 94) in private kindergartens. Additionally, 61.60% (*n* = 377) were employed in urban areas and 38.40% (*n* = 235) in rural areas.

Sample 2 comprised 612 kindergarten teachers, with a mean age of 34.84 years (*SD* = 8.27; range = 20–60) and a mean teaching experience of 10.97 years (*SD* = 8.84; range = 1–40). Regarding educational attainment, 34.00% (*n* = 208) held a junior college degree or lower, whereas 66.00% (*n* = 404) held a bachelor's degree or higher. In terms of kindergarten type, 87.90% (*n* = 538) worked in public kindergartens and 12.10% (*n* = 74) in private kindergartens. Additionally, 67.60% (*n* = 414) were employed in urban areas and 32.40% (*n* = 198) in rural areas. Chi-square tests showed that the two subsamples differed significantly only in urban–rural location (*p* = 0.032), but not in educational attainment or kindergarten type (*ps* > 0.05). Independent-samples *t* tests showed no significant differences in age, years of teaching experience, AJC, or AvJC (*ps* > 0.05).

To assess the test–retest reliability of the KTJCS, 139 kindergarten teachers were randomly selected from the full sample for a follow-up assessment three months after the initial survey. The retest sample had a mean age of 36.90 years (*SD* = 9.37; range = 22–54) and a mean teaching experience of 12.78 years (*SD* = 10.77; range = 1–34). Regarding educational attainment, 23.00% (*n* = 32) held a junior college degree or lower, whereas 77.00% (*n* = 107) held a bachelor's degree or higher. In terms of kindergarten type, 82.70% (*n* = 115) worked in public kindergartens and 17.30% (*n* = 24) in private kindergartens.

### Measures

#### Kindergarten Teacher Job Crafting Scale (KTJCS)

Based on the qualitative themes identified in Study 1, an initial item pool was developed to assess kindergarten teachers' job crafting. The preliminary scale comprised two subscales: AJC and AvJC. The AJC subscale included 27 items across three dimensions: task crafting (11 items), relational crafting (9 items), and cognitive crafting (7 items). The AvJC subscale included 21 items across three dimensions: task crafting (7 items), relational crafting (7 items), and cognitive crafting (7 items). All items were rated on a 5-point Likert scale ranging from 1 (never) to 5 (always). As the scale was originally developed in Chinese, the item wording was translated into English and then back-translated into Chinese to ensure accuracy and consistency.

#### Broad Job Crafting Scale (BJCS)

The Broad Job Crafting Scale [39] was used to assess convergent validity. The original items were translated into Chinese and then back-translated into English to ensure linguistic equivalence. The four items (e.g., “I adjust my work to make myself feel better”) were rated on a 5-point Likert scale ranging from 1 (strongly disagree) to 5 (strongly agree). In the present study, Cronbach's* α* and composite reliability (CR) were 0.943 and 0.945, respectively.

#### Work engagement scale

Work engagement was assessed using the 9-item Utrecht Work Engagement Scale (UWES; [29]). The scale comprises three dimensions: vigor, dedication, and absorption. The items (e.g., “I feel energetic at work”) were rated on a 7-point Likert scale ranging from 1 (strongly disagree) to 7 (strongly agree). In the present study, Cronbach's *α* and CR were 0.942 and 0.950, respectively.

#### Work meaning scale

Work meaning was assessed using the scale developed by Spreitzer [34]. The three items (e.g., “The work I do is very meaningful to me”) were rated on a 5-point Likert scale ranging from 1 (strongly disagree) to 5 (strongly agree). In the present study, Cronbach's *α* and CR were 0.953 and 0.956, respectively.

#### Job satisfaction scale

Job satisfaction was assessed using the job satisfaction subscale of the Michigan Organizational Assessment Questionnaire [4]. The three items (e.g., “Overall, I am satisfied with my job”) were rated on a 7-point Likert scale ranging from 1 (strongly disagree) to 7 (strongly agree). In the present study, Cronbach's *α* and CR were 0.704 and 0.738, respectively.

### Analytic strategy

Data were analyzed using SPSS 21.0 and Mplus 8.3. In Sample 1, SPSS 21.0 was used for item analysis and exploratory factor analysis (EFA). Item analysis included extreme-group comparisons and homogeneity analysis. For the extreme-group comparisons, participants were divided into the top 27% (high-score group) and bottom 27% (low-score group) based on each subscale total score. Independent-samples *t* tests were conducted for each item to assess item discrimination, and items showing significant between-group differences were considered to have adequate discrimination [33]. For homogeneity analysis, Pearson correlation coefficients between each item and the total subscale score were calculated, with coefficients above 0.40 considered acceptable [50].

EFA was then conducted separately for the AJC and AvJC subscales to examine their underlying factor structures. Principal axis factoring with oblique rotation was adopted. Factor retention was guided by eigenvalues greater than 1 [21], scree plots, and parallel analysis. To obtain a parsimonious and stable factor structure, items were sequentially removed based on the following criteria: (1) factor loadings below 0.40 on the intended factor [9, 46]; (2) cross-loadings above 0.40 on two or more factors [30, 33]; (3) communalities below 0.50 [44]; and (4) inadequate representation of the core content of the intended dimension or unclear conceptual alignment with that dimension [50].

In Sample 2, CFA was conducted in Mplus 8.3 to test the factor structure suggested by the EFA. Models were estimated using robust maximum likelihood estimation (MLR). Model fit was evaluated using multiple indices. Model fit was considered acceptable if RMSEA and SRMR values were below 0.08 and CFI and TLI values exceeded 0.90 [13]. Alternative models were compared with the hypothesized second-order model. Following Vianello et al. [40], ΔCFI values greater than 0.010 and ΔRMSEA values greater than 0.015 were taken to indicate meaningful differences in model fit. Convergent validity was assessed in two ways. First, Pearson correlation coefficients were calculated between the total and dimensional scores of the KTJCS and the total score of the BJCS [39]. Second, average variance extracted (AVE) values were examined, with values above 0.50 indicating adequate convergent validity. Discriminant validity was evaluated by comparing the square root of the AVE for each factor with its correlations with other factors. It was considered acceptable when the square root of the AVE exceeded the inter-factor correlations. Criterion-related validity was examined through correlations between the KTJCS and work engagement, work meaning, and job satisfaction.

Reliability was evaluated using Cronbach's *α*, CR, and test–retest reliability. Cronbach's *α* and CR values of 0.70 or above were considered indicative of acceptable internal consistency [41]. Measurement invariance across kindergarten type and urban–rural location was tested through sequential comparisons of configural, metric, scalar, and strict invariance models. Following Cheung and Rensvold [6], ΔCFI < 0.010 and ΔRMSEA < 0.015 were taken to support measurement invariance across groups.

## Results

### Item analysis

Extreme-group comparisons showed that all items in the AJC and AvJC subscales significantly distinguished between the high- and low-score groups. For the AJC subscale, *t* values ranged from 16.278 to 31.757, and for the AvJC subscale, *t* values ranged from 11.322 to 39.596. All comparisons were statistically significant (*p* < 0.001). Item–total correlations ranged from 0.584 to 0.833 for AJC and from 0.483 to 0.891 for AvJC, with all coefficients exceeding 0.40. These results indicated that all initial items demonstrated acceptable item discrimination and item homogeneity.

### Exploratory factor analysis

**Fig. 1:**
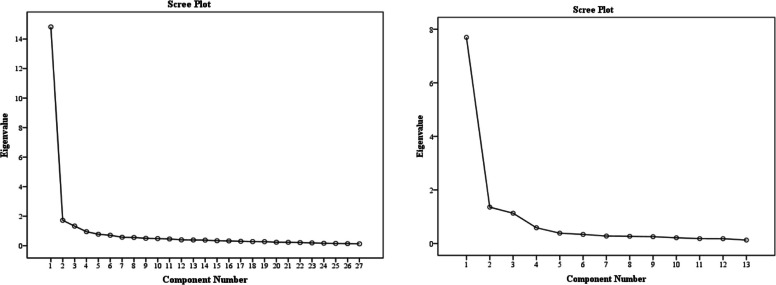
Scree plots for the AJC and AvJC subscales in the exploratory factor analyses (left: AJC; right: AvJC)

The Kaiser–Meyer–Olkin (KMO) values indicated excellent sampling adequacy for both subscales (AJC = 0.962; AvJC = 0.964), and Bartlett's test of sphericity was significant (AJC: *χ*^2^(351) = 13,910.511, *p* < 0.001; AvJC: *χ*^2^(210) = 15,068.202, *p* < 0.001), supporting the suitability of the data for factor analysis. Initial EFA results suggested a three-factor solution with eigenvalues greater than 1 for both subscales. This solution was also supported by the scree plots (see Fig. 1). For AJC, the eigenvalues were 14.823, 1.719, and 1.328, accounting for 66.183% of the total variance. For AvJC, the eigenvalues were 13.672, 1.483, and 1.216, accounting for 77.953% of the total variance.

Based on the predefined criteria, 9 items were removed from the AJC subscale and 8 from the AvJC subscale. For the AJC subscale, the removed items comprised 1 item with a low factor loading, 5 with high cross-loadings, 1 with low communality, and 2 with inadequate content representativeness or unclear conceptual alignment. For the AvJC subscale, the removed items comprised 1 item with a low factor loading, 5 with high cross-loadings, and 2 with inadequate content representativeness or unclear conceptual alignment. After item reduction, each dimension retained at least 4 items. The final scale comprised 16 AJC and 13 AvJC items. Parallel analysis also supported the retention of 3 factors for both subscales.

The final EFA confirmed the suitability of the data for factor analysis (AJC: KMO = 0.938, *χ*^2^(120) = 6,443.603, *p* < 0.001; AvJC: KMO = 0.940, *χ*^2^(78) = 6,720.465, *p* < 0.001). For the AJC subscale, the eigenvalues were 8.373, 1.481, and 1.165, accounting for 68.862% of the total variance. For the AvJC subscale, the eigenvalues were 7.643, 1.396, and 1.132, accounting for 78.242% of the total variance. All retained items loaded on their intended factors, with primary loadings ranging from 0.598 to 0.897 (see Table 1). The final factor structure was generally consistent with the interview findings, although two items initially classified as relational crafting were reassigned to task crafting.

**Table 1:** The Factor Loadings of the Final Explanatory Factor Analysis (*n* = 612)

	**AJC**			**AvJC**			**communality**	**Origin theme**
	**ATC**	**ARC**	**ACC**	**AvTC**	**AvRC**	**AvCC**		
A1. I proactively adjust the activity content and methods based on the children's interests	0.777						0.543	ATC
A2. I proactively adjust the layout or decoration of the classroom play areas and theme walls	0.768						0.574	ATC
A3. I actively seek various teaching materials to enrich daily teaching	0.865						0.671	ATC
A4. I make efforts to improve the quality of communication with the children	0.824						0.645	ARC
A5. I make efforts to understand the issues that parents are concerned about to facilitate communication	0.614						0.606	ARC
A9. I increase emotional communication with the children to establish trust	0.598						0.595	ATC
A6. I proactively seek feedback and suggestions from the principal about my work performance		0.799					0.639	ARC
A7. I proactively seek work advice from colleagues		0.758					0.692	ARC
A10. I proactively seek support and encouragement from the principal		0.827					0.596	ARC
A11. I proactively communicate and collaborate with colleagues		0.628					0.642	ARC
A16. I realize that my work is important for social development			0.615				0.591	ACC
A17. I regard early childhood education as my career			0.867				0.634	ACC
A19. I think about how early childhood education can improve my quality of life			0.649				0.427	ACC
A21. I see early childhood education as a way to achieve self-realization			0.897				0.741	ACC
A24. I realize that my work in early childhood education enhances my overall well-being			0.739				0.629	ACC
A26. I am aware of my mission and responsibilities as a kindergarten teacher			0.737				0.663	ACC
D1. I try to avoid changing my routine teaching activities and methods				0.686			0.405	AvTC
D2. I postpone completing tasks that I am not good at				0.811			0.704	AvTC
D4. I try not to make difficult decisions in my work				0.860			0.771	AvTC
D6. I spend less time on work tasks that I am not interested in				0.826			0.760	AvTC
D3. I try to avoid in-depth interactions with colleagues with whom I have strained relationships					0.866		0.846	AvRC
D5. I try to reduce interactions with colleagues who I do not get along with					0.869		0.687	AvRC
D7. I try to minimize conversations with leaders who may cause conflicts					0.812		0.777	AvRC
D16. I try to stay away from colleagues who affect my work mood					0.759		0.593	AvRC
D8. I rarely think about work tasks that cause stress						0.707	0.686	AvCC
D9. I try to avoid thinking deeply about work tasks I don't like						0.887	0.750	AvCC
D17. I try to avoid thinking about complex or difficult tasks						0.893	0.806	AvCC
D19. I rarely imagine working with difficult colleagues						0.842	0.742	AvCC
D20. I try to avoid thinking about tasks that are not helpful to my career development						0.880	0.797	AvCC

Scoring instructions: Scores for each first-order dimension are computed by averaging the corresponding item responses. The second-order scores for Approach Job Crafting and Avoidance Job Crafting are computed by averaging the scores of their three respective first-order dimensions. Higher scores indicate higher levels of the corresponding type of job crafting

*Abbreviations: AJC* Approach Job Crafting, *ATC* Approach Task Crafting, *ARC* Approach Relational Crafting, *ACC* Approach Cognitive Crafting, *AvJC* Avoidance Job Crafting, *AvTC* Avoidance Task Crafting, *AvRC* Avoidance Relational Crafting, *AvCC* Avoidance Cognitive Crafting

### Confirmatory factor analysis

**Fig. 2:**
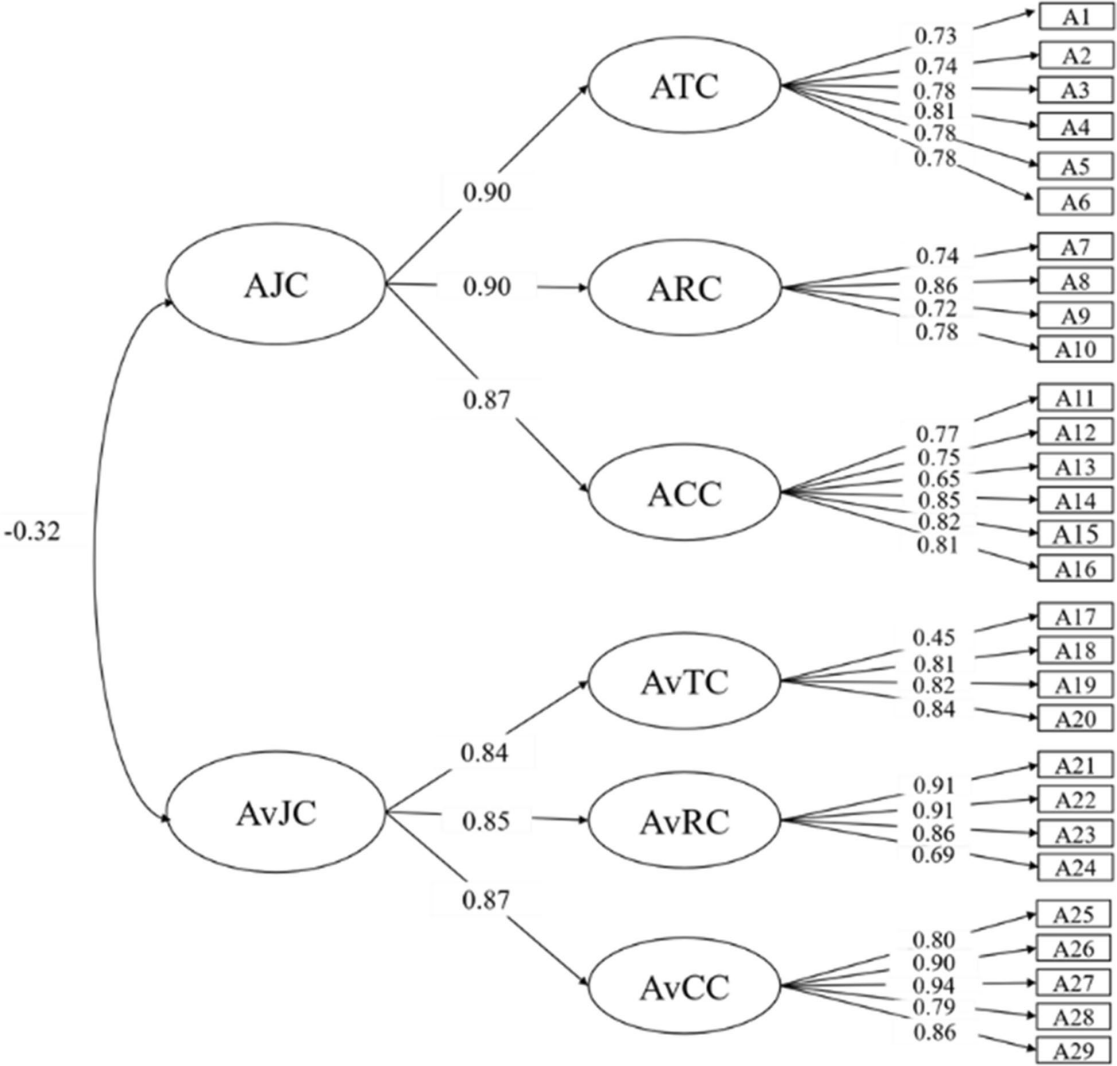
Second-order model of the KTJCS. Notes: All numbers in Figure represent standardized factor loadings, and all are significant at *p* < 0.001. Based on the results of exploratory factor analysis, some items were deleted, and the remaining items were recoded. Abbreviations: AJC, Approach Job Crafting; ATC, Approach Task Crafting; ARC, Approach Relational Crafting; ACC, Approach Cognitive Crafting; AvJC, Avoidance Job Crafting; AvTC, Avoidance Task Crafting; AvRC, Avoidance Relational Crafting; AvCC, Avoidance Cognitive Crafting

The model fit results indicated that the unidimensional, first-order two-factor, and first-order three-factor models exhibited poor fit, whereas the bifactor and correlated first-order factor models showed improved fit (see Table 2). The second-order model showed the best overall fit among the tested models. In this model, all items loaded significantly on their respective first-order latent factors (*p* < 0.001), with standardized factor loadings ranging from 0.455 to 0.941 (see Fig. 2).

**Table 2:** Results of the Confirmatory Factor Analysis (*n* = 612)

Model	*χ*^2^ (*df*)	*χ*^2^/*df*	CFI	TLI	RMSEA	90%CI of RMSEA	SRMR
Unidimensional Model	5434.831 (377)	14.416	0.440	0.397	0.148	[0.145 0.152]	0.196
First-Order Two-Factor Model	2221.951 (376)	5.909	0.796	0.779	0.090	[0.086 0.093]	0.065
First-Order Three-Factor Model	4982.667 (374)	13.323	0.490	0.446	0.142	[0.138 0.145]	0.202
bifactor model	1351.551 (348)	3.884	0.889	0.870	0.069	[0.065 0.073]	0.158
correlated first-order factors Model	1167.497 (362)	3.225	0.937	0.930	0.060	[0.056 0.064]	0.045
Second-Order Model	889.822 (370)	2.405	0.942	0.937	0.048	[0.044 0.052]	0.050

The unidimensional model specified all items loading on a single latent job crafting factor. The first-order two-factor model specified all items loading on two correlated first-order factors representing AJC and AvJC. The first-order three-factor model specified all items loading on three correlated first-order factors representing task, relational, and cognitive crafting. The bifactor model specified one general job crafting factor and six specific first-order factors representing the six dimensions of job crafting. The correlated first-order factors model specified six correlated first-order factors: approach task crafting, approach relational crafting, approach cognitive crafting, avoidance task crafting, avoidance relational crafting, and avoidance cognitive crafting. The second-order model specified six first-order factors loading onto two second-order factors, AJC and AvJC

### Convergent and discriminant validity

AVE values ranged from 0.561 to 0.738, all exceeding the recommended threshold of 0.50 and indicating satisfactory convergent validity ([11]; see Table 3). AJC total and dimensional scores showed moderate positive correlations with the BJCS total score, whereas AvJC total and dimensional scores showed significant negative correlations with the BJCS total score (see Table 4). These findings provide additional convergent evidence for AJC and further support the conceptual distinction between AJC and AvJC. The square roots of the AVE values for all dimensions exceeded their correlations with other dimensions, indicating adequate discriminant validity (see Table 3).

**Table 3:** Correlations and the AVE square root for each factor (*n* = 612)

	1	2	3	4	5	6
1 ATC	0.771(0.603)					
2 ARC	0.707^***^	0.777(0.604)				
3 ACC	0.701^***^	0.677^***^	0.780(0.609)			
4 AvTC	−0.195^***^	−0.193^***^	−0.241^***^	0.749(0.561)		
5 AvRC	−0.096^*^	−0.169^***^	−0.190^***^	0.599^***^	0.849(0.721)	
6 AvCC	−0.184^***^	−0.220^***^	−0.292^***^	0.625^***^	0.705^***^	0.859(0.738)

The value in the diagonal cell is the square root of AVE for each factor, and the value in parentheses is the AVE of each factor

^***^p < 0.001

**Table 4:** Correlations between the KTJCS and job satisfaction, work engagement, and work meaning (*n* = 612)

	*M* ± *SD*	1	2	3	4	5	6	7	8	9
1 AJC	4.142 ± 0.582	1								
2 ATC	0.423 ± 0.601	0.901^***^	1							
3 ARC	3.934 ± 0.726	0.867^***^	0.707^***^	1						
4 ACC	4.184 ± 0.670	0.905^***^	0.701^***^	0.677^***^	1					
5 AvJC	2.170 ± 0.819	−0.256^***^	−0.180^***^	−0.223^***^	−0.278^***^	1				
6 AvTC	2.205 ± 0.856	−0.237^***^	−0.195^***^	−0.193^***^	−0.241^***^	0.824^***^	1			
7 AvRC	2.272 ± 1.021	−0.170^***^	−0.096^*^	−0.169^***^	−0.190^***^	0.884^***^	0.599^***^	1		
8 AvCC	2.060 ± 0.922	−0.263^***^	−0.184^***^	−0.220^***^	−0.292^***^	0.907^***^	0.625^***^	0.705^***^	1	
9 BJC	4.282 ± 0.644	0.646^***^	0.531^***^	0.536^***^	0.647^***^	−0.189^***^	−0.173^***^	−0.131^**^	−0.190^***^	1
10 JS	5.893 ± 1.003	0.475^***^	0.362^***^	0.341^***^	0.539^***^	−0.410^***^	−0.350^***^	−0.332^***^	−0.390^***^	0.531^***^
11 WE	5.845 ± 1.101	0.563^***^	0.432^***^	0.430^***^	0.619^***^	−0.393^***^	−0.331^***^	−0.311^***^	−0.395^***^	0.578^***^
12 WM	4.263 ± 0.745	0.554^***^	0.428^***^	0.423^***^	0.606^***^	−0.321^***^	−0.265^***^	−0.254^***^	−0.331^***^	0.544^***^

*Abbreviations: AJC* Approach Job Crafting, *ATC* Approach Task Crafting, *ARC* Approach Relational Crafting, *ACC* Approach Cognitive Crafting, *AvJC* Avoidance Job Crafting, *AvTC* Avoidance Task Crafting, *AvRC* Avoidance Relational Crafting, *AvCC* Avoidance Cognitive Crafting, *BJC* Broad Job Crafting, *JS* Job Satisfaction, *WE* Work Engagement, *WM* Work Meaning

^***^p < 0.001

### Criterion-related validity

Prior to the validity analyses, a single-factor CFA model including AJC, AvJC, job satisfaction, work engagement, and work meaning was tested to assess common method bias. The model showed poor fit, *χ*^2^(902) = 9,707.863, *χ*^2^/*df* = 10.762, SRMR = 0.162, RMSEA = 0.126, CFI = 0.528, and TLI = 0.505, suggesting that common method bias was unlikely to pose a serious threat to the study findings.

AJC total and dimensional scores were positively correlated with job satisfaction, work engagement, and work meaning, whereas AvJC total and dimensional scores were significantly negatively correlated with these outcomes (see Table 4). Additional analyses were conducted using a latent-variable model to account for the relatively lower reliability of the job satisfaction scale. The results indicated that AJC was positively associated with job satisfaction (*r* = 0.634, SE = 0.041, 90% CI [0.553, 0.715]), whereas AvJC was negatively associated with job satisfaction (*r* = −0.451, SE = 0.039, 90% CI [−0.528, −0.374]).

### Reliability

For the AJC subscale, Cronbach's *α* coefficients ranged from 0.849 to 0.941, CR values ranged from 0.858 to 0.948, and test–retest reliability coefficients ranged from 0.720 to 0.813. For the AvJC subscale, Cronbach's *α* coefficients ranged from 0.811 to 0.936, CR values ranged from 0.830 to 0.964, and test–retest reliability coefficients ranged from 0.706 to 0.766 (see Table 5). All coefficients exceeded the recommended threshold of 0.70, indicating acceptable reliability for the KTJCS.

**Table 5:** Reliability analysis of the KTJCS

	AJC	ATC	ARC	ACC	AvJC	AvTC	AvRC	AvCC
Cronbach's *α* Coefficient (*n* = 612)	0.941	0.896	0.849	0.897	0.936	0.811	0.905	0.931
CR Coefficient (*n* = 612)	0.948	0.897	0.858	0.903	0.964	0.830	0.911	0.933
Retest reliability (*n* = 139)	0.813	0.776	0.720	0.734	0.766	0.743	0.757	0.706

### Measurement invariance

For both kindergarten type and urban–rural location, the configural, metric, scalar, and strict invariance models showed acceptable fit (see Table 6). Comparisons of adjacent models showed that all ΔCFI values were below 0.010 and all ΔRMSEA values were below 0.015, supporting measurement invariance across groups.

**Table 6:** Measurement invariance tests of the KTJCS across kindergarten type and region (*N* = 1224)

	Model	*χ*^2^	*df*	*χ*^2^/*df*	CFI	TLI	SRMR	RMSEA	90% CI of RMSEA	ΔCFI	ΔRMSEA
kindergarten type											
AJC	M1	760.214	202	3.763	0.926	0.912	0.045	0.067	[0.062, 0.072]		
	M2	779.193	215	3.624	0.925	0.917	0.049	0.065	[0.061, 0.070]	0.001	0.003
	M3	813.970	228	3.570	0.923	0.918	0.052	0.065	[0.060, 0.070]	0.002	< 0.001
	M4	875.077	244	3.586	0.917	0.918	0.060	0.065	[0.060, 0.070]	0.006	< 0.001
AVJC	M1	349.102	124	2.815	0.983	0.978	0.027	0.054	[0.048, 0.061]		
	M2	352.540	134	2.631	0.983	0.980	0.028	0.052	[0.045, 0.058]	< 0.001	0.002
	M3	365.776	144	2.540	0.983	0.981	0.028	0.050	[0.044, 0.057]	< 0.001	0.002
	M4	477.118	157	3.39	0.975	0.975	0.032	0.058	[0.052, 0.064]	0.008	0.003
urban–rural location											
AJC	M1	750.508	202	3.715	0.923	0.909	0.047	0.067	[0.062, 0.072]		
	M2	766.889	215	3.567	0.923	0.914	0.050	0.065	[0.060, 0.070]	< 0.001	0.002
	M3	800.890	228	3.513	0.920	0.916	0.050	0.064	[0.059, 0.069]	0.003	0.001
	M4	825.933	244	3.385	0.919	0.920	0.060	0.062	[0.058, 0.067]	0.001	0.002
AVJC	M1	356.931	124	2.878	0.982	0.977	0.027	0.055	[0.049, 0.062]		
	M2	374.348	134	2.793	0.981	0.978	0.031	0.054	[0.048, 0.061]	0.001	0.001
	M3	393.538	144	2.733	0.981	0.979	0.033	0.053	[0.047, 0.060]	< 0.001	0.001
	M4	456.105	157	2.905	0.977	0.977	0.034	0.056	[0.050, 0.062]	0.004	0.003

M1 = Configural invariance model; M2 = Weak invariance model; M3 = Strong invariance model; M4 = Strict invariance model

*Abbreviations; df* Degrees of freedom, *CFI* Comparative fit index, *TLI* Tucker-Lewis index, *SRMR* standardized root mean squared residual, *RMSEA* Root mean square error of approximation, *CI* Confidence interval

## Discussion

The present study developed and validated the KTJCS as an occupation-specific measure of job crafting in kindergarten teachers in China. The results confirmed two subscales, AJC and AvJC, each encompassing task, relational, and cognitive crafting. The scale demonstrated satisfactory reliability, validity, and measurement invariance across groups, supporting its use in future research on job crafting in early childhood education.

The findings showed that AJC was positively associated with work engagement, job satisfaction, and work meaning, whereas AvJC was negatively associated with these outcomes. Consistent with prior research [7, 16, 17], AJC facilitates resource acquisition, promotes active work involvement, and supports the construction of positive work meaning, thereby contributing to more sustained and favorable work experiences. By contrast, AvJC primarily minimizes exposure to demanding and adverse work conditions. While this may alleviate immediate strain, it concurrently constrains opportunities for resource gain, meaningful engagement, and identity-relevant meaning making. Consequently, AvJC shows weaker associations with sustained positive work experiences. These findings support the distinction between AJC and AvJC in kindergarten teaching.

The results revealed a multidimensional structure of job crafting among kindergarten teachers, comprising task, relational, and cognitive forms nested within both AJC and AvJC. Extending prior integrated perspectives [18], our findings differentiate behavioral crafting into distinct task and relational dimensions, while treating cognitive crafting as a unified dimension reflecting the cognitive redefinition of work content and identity. Unlike prior models that impose a rigid resource and demand dichotomy or reduce cognitive crafting to demand management, our structure captures resource- and demand-related characteristics through approach- and avoidance-motivated enactments across all three dimensions. This approach preserves the conceptual richness of the role-based perspective while incorporating the functional dynamics emphasized by the resource perspective, aligning with the specific occupational context of Chinese kindergarten teachers.

These structural findings also have important implications for score interpretation. The findings support the conceptualization of AJC and AvJC as two distinct second-order constructs that may serve as the primary basis for interpretation. Depending on specific research objectives, more fine-grained differences may be examined through the first-order dimensions of task, relational, and cognitive crafting. This scoring strategy may enhance interpretive clarity and provide a more targeted analytical framework for future research.

Beyond structural differentiation, these dimensions exhibited context-specific content that distinguished kindergarten teachers' job crafting from their general conceptualizations. For AJC, task crafting extends beyond modifying task arrangements to include professional interactions with children and parents, indicating that sustained interpersonal engagement constitutes a core work task. Relational crafting involves actively shaping interpersonal relationships, with managing relationships with leaders being equally salient, unlike prior research that focused mainly on colleagues [25]. Consistent with its established definition [43], cognitive crafting is reflected in the positive reframing of work meaning. In the kindergarten context, this reframing supports sustained engagement amid emotionally demanding work, extending the integrated perspective on cognitive crafting. For AvJC, avoidance-oriented strategies largely align with prior conceptualizations. However, in the kindergarten context, they may serve a particularly salient short-term adaptive function, given the high emotional and relational demands of the work.

The KTJCS demonstrated satisfactory psychometric properties and can serve as a useful tool for research and practice in early childhood education. In research contexts, the scale can be used to examine developmental patterns and dynamic changes of job crafting among kindergarten teachers and model its associations with relevant individual and contextual factors. In practice, the KTJCS enables the identification of teachers' job crafting patterns and potential risk signals. Stronger tendencies toward AvJC may indicate high work pressure, insufficient resources, or a mismatch between job demands and available support. Continuous assessment of job crafting allows administrators to monitor teachers' work adaptation and well-being and evaluate the effectiveness of interventions aimed at promoting more adaptive, approach-oriented job crafting. These applications contribute to teachers' professional development and the quality of early childhood education.

### Limitations and future research

This study has some limitations. First, the data were based primarily on teachers' self-reports collected at a single time point. Although this method captures subjective job crafting experiences, it may increase the risk of common method variance and inflate the observed associations among the variables. Future research could use multi-source data and observational or diary methods to test these relationships more rigorously. Second, the criterion variables were work engagement, job satisfaction, and work meaning, which are conceptually more aligned with AJC than with AvJC. The current criterion-related validity evidence provides stronger support for AJC, whereas the external validity of AvJC remains less fully examined. Future research could include variables more directly related to AvJC, such as stress, emotional exhaustion, withdrawal behavior, and avoidant coping. Third, although the sample included teachers from both northern and southern China, the participants were drawn only from Guangdong and Jilin provinces. Findings may not be fully generalizable to kindergarten teachers across China, and the stability of the factor structure in other regional and sociocultural contexts remains to be examined. Future research could further examine the scale's cross-regional applicability using diverse samples. Finally, the study relied on cross-sectional data and therefore could not establish the directionality of associations among variables or examine how these relationships develop over time. Future research could adopt longitudinal and intervention designs to further examine the stability of the observed relationships as well as the predictive validity and practical usefulness of the scale.

## Conclusion

This study developed and validated the KTJCS, which comprises two subscales, AJC (16 items) and AvJC (13 items), each of which includes task, relational, and cognitive crafting dimensions. The findings support the scale's reliability, validity, and cross-group measurement invariance, indicating that the KTJCS is a reliable and valid measure of kindergarten teachers' job crafting. Grounded in kindergarten teachers' real work experiences, the KTJCS provides a foundation for future research and has potential value for teacher development and interventions.

## Data Availability

The data presented in this study are available on request from the first authors Xiaoqing Lin (linxq799@nenu.edu.cn).
